# Mechanisms of increased bioavailability through amorphous solid dispersions: a review

**DOI:** 10.1080/10717544.2019.1704940

**Published:** 2019-12-30

**Authors:** Andreas Schittny, Jörg Huwyler, Maxim Puchkov

**Affiliations:** a Department of Pharmaceutical Sciences, Division of Pharmaceutical Technology, University of Basel, Basel, Switzerland;; b Department of Biomedicine, Division of Clinical Pharmacology and Toxicology, University Hospital Basel and University of Basel, Basel, Switzerland

**Keywords:** Amorphous solid dispersion, excipients, poorly water-soluble drugs, oral bioavailability, dissolution, drug absorption

## Abstract

Amorphous solid dispersions (ASDs) can increase the oral bioavailability of poorly soluble drugs. However, their use in drug development is comparably rare due to a lack of basic understanding of mechanisms governing drug liberation and absorption *in vivo*. Furthermore, the lack of a unified nomenclature hampers the interpretation and classification of research data. In this review, we therefore summarize and conceptualize mechanisms covering the dissolution of ASDs, formation of supersaturated ASD solutions, factors responsible for solution stabilization, drug uptake from ASD solutions, and drug distribution within these complex systems as well as effects of excipients. Furthermore, we discuss the importance of these findings on the development of ASDs. This improved overall understanding of these mechanisms will facilitate a rational ASD formulation development and will serve as a basis for further mechanistic research on drug delivery by ASDs.

## 1. Introduction

Even though poorly soluble drug candidates constitute the largest amount of drug candidates in the development, they suffer from the highest attrition rates (Lipp, 2013), frequently due to low bioavailability (Waring et al., 2015). A potential solution is drug delivery in the form of amorphous solid dispersions (ASDs) (Padden et al., 2011). In the last decades, ASDs have been researched with increasing interest, as showed in a recent literature and patent analysis: In both, academia and industry, an exponential increase of articles and patents was observed (Zhang et al., 2018).

Various definitions of ASDs have been used, often taking into account underlying physicochemical properties (e.g. being eutectic), the absence or presence of crystallinity, or the thermodynamic vs. the kinetic stability of the system. However, in the context of pharmaceutical drug delivery, a definition that is also used in this article has prevailed: ASDs are systems in which an active pharmaceutical ingredient (API) is embedded largely amorphously into a solid matrix, often consisting of polymers (Huang & Dai, 2014).

The use of ASDs in oral drug delivery has shown to enhance *in vitro* performance as well as *in vivo* bioavailability in animals (Yu et al., 2013; Fule et al., 2015, 2016; Agrawal et al., 2016; Kate et al., 2016; Mitra et al., 2016; Xia et al., 2016; Zhang et al., 2016; Knopp et al., 2018; Liu et al., 2018) and in humans (Six et al., 2005; Weiss et al., 2009; Moes et al., 2011; Aboelwafa & Fahmy, 2012; Krishna et al., 2012; Marchetti et al., 2012; Zayed et al., 2012; Othman et al., 2012a,b; Moes et al., 2013; Prasannaraju et al., 2013; Shah et al., 2013; Anon, 2014). An overall statistically positive effect of ASD on bioavailability was measured in a recent meta-analysis (Fong et al., 2017). In addition, ASDs show advantages over other formulation strategies of poorly soluble drugs, such as solubilization in micelles (Miller et al., 2011), self-emulsifying drug delivery systems or cyclodextrins (Dahan et al., 2010; Park et al., 2018), or cosolvents (Miller et al., 2012b). However, an analysis based on 40 research papers showed that 18% of ASD formulations decreased or did not increase bioavailability *in vivo* (animals and humans) (Newman et al., 2012). Among the marketed drugs, out of 3732 registered drug products (2019) (Wishart et al., 2018), only 24 were ASD formulations (2015) (Newman, 2015). These constitute roughly 0.6% of drugs on the market, indicating that ASDs seem not to be used to their full potential in today’s drug development.

Reasons for this could be that ASDs are more complex systems (Park, 2015) compared to standard drug formulations: At first, the ability of an API to form an ASD with a specific polymer is not guaranteed, as the process of mixing or dissolution, e.g. in a molten state, of an API in a polymer might not be favorable from a thermodynamic point of view; therefore, ASDs, if formed under such conditions, are either unstable or cannot be manufactured. Second, the production involves complex processes such as hot-melt extrusion. Once produced, stability for suitable shelf life is still a vital issue, as crystallization can occur post-production. These hurdles result in high development costs without a guarantee of an increased bioavailability. To enhance the mechanistic understanding of increased bioavailability through ASDs, research activities are ongoing. However, this process is far from being entirely understood (Tho et al., 2010; Park, 2015; Fong et al., 2017). As decisions on the further development or dropout of drug candidates are made as early as possible in today’s drug development process (Paul et al., 2010), estimating the potential of an API to be delivered as ASD becomes crucial to support the decision to further develop poorly soluble drug candidates. In this respect, predictive tools and models, and therefore, mechanistic understanding for ASD formulations, are essential to reduce the attrition rate of poorly soluble drug candidates. Famous examples of such predictive possibilities are the biopharmaceutical classification system (Amidon et al., 1995) or Lipinski’s ‘Rule of Five’ (Fischer & Breitenbach, 2013). Such methods and insights allow for feasibility estimations without or only a limited number of experiments. For ASDs, such approaches are minimal.

In this review, we provide a summary of reports currently available that elucidate underlying mechanisms of increased bioavailability based on theoretical considerations as well as on experimental data *in vitro*, *in vivo* (including humans) and conceptualize them into a common context. We propose mechanisms of ASD dissolution, supersaturation stabilization, drug uptake, and API distribution within the complex dissolved system, focusing on polymeric ASDs with or without additional excipients. Furthermore, we propose a unified nomenclature to facilitate the interpretation and classification of research data. We discuss the implications of our observations on ASD formulation development. We thus aim to contribute to better understanding of mechanisms contributing to increased oral bioavailability through ASDs and rationalized ASD formulation development.

## 2. Literature research results and their use in this article

We performed literature research based on standard literature research engines. We use the results of the individual articles in a nonsystematic way, aiming to highlight their most important outcomes and their relations to other articles. It turned out that the larger part of research papers on ASDs do not focus on the mechanisms behind increased bioavailability, but rather look at the development of ASDs for individual drugs. Also, there seems to be no consent on specific wordings (e.g. drug-rich particles) as such terms were used differently by various authors. In this review, where necessary we therefore introduced the nomenclature to enable for a clear comparison between different articles.

## 3. Conceptual prerequisites for bioavailability of APIs from ASDs

**Figure 1.**
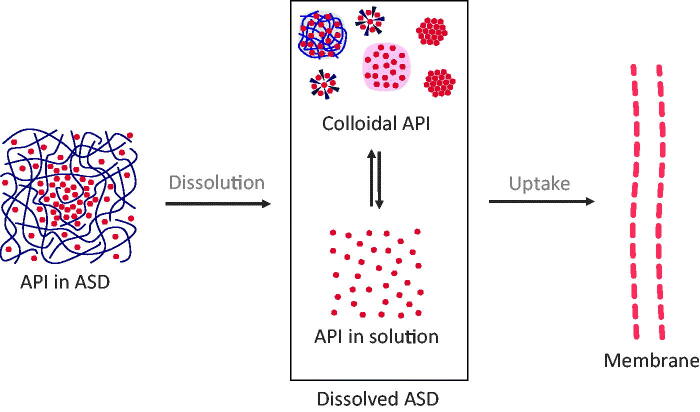
Basic concept of drug uptake from ASDs. From the solid state of ASDs (drug-rich particles of pure drug (ALPS), drug-rich particles containing polymers, micelles, and crystals), a complex mixture of API in solution and colloidal API emerges, from which the drug uptake through the intestinal membrane is induced.

To structure this review, we follow the general mechanism for drug uptake from conventional formulations as a starting point and extended it to the ASDs related situation by reviewing reports investigating mechanisms of drug uptake from ASDs (Figure 1). Upon contact of ASDs with the aqueous medium, spontaneous dissolution into classical solution (molecularly dissolved API) takes place. For ASDs however, there are further states of dissolved API known, such as drug-rich particles, micelles, or suspensions of crystals (not molecularly dissolved). We refer to the whole multitude of those states as the colloidal system formed upon the dissolution of ASDs. From the dissolved form of the ASD, an uptake of an API through the intestinal wall is induced. Overall, the uptake of API from solid ASDs therefore is a complex, multi-stage process, which is reviewed in the following sections:

- Dissolution from solid ASDs to dissolved ASDs (Section 5);
- Dissolved ASDs: described states and their stabilization (Section 6);
- Drug uptake from dissolved ASDs (Section 7);
- Equilibria and API distribution within the dissolved ASDs during dissolution and uptake (Section 8).

In recent years, the impressive work of Taylor et al. contributed majorly to today’s understanding of increased bioavailability of ASDs. For details concerning the physicochemical mechanisms of supersaturated solutions and implications for drug uptake, the reader is referred to the corresponding review (Taylor & Zhang, 2016). In this work, discussions are often based on insight gained by her research group.

## 4. Physicochemical background: drug solubility, supersaturation, and solubilization

**Figure 2.**
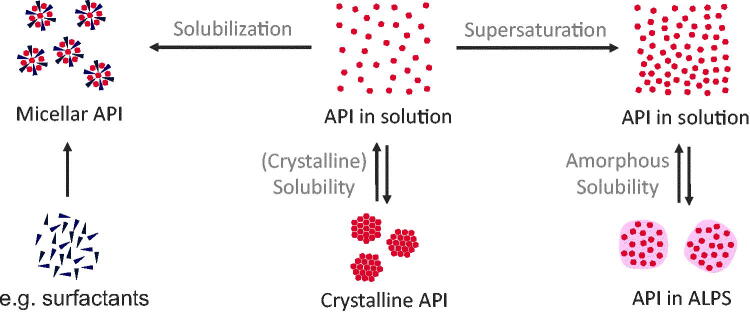
Classification of physicochemical concepts of solubility, supersaturation, and solubilization. Two equilibria of API in solution can be differentiated: (1) the equilibrium between crystalline API and API in solution is referred to the crystalline solubility and (2) the equilibrium between API in amorphous liquid phase separation (ALPS) and API in solution. Compared to equilibrium 1, equilibrium 2 shows a higher concentration of molecularly dissolved drug (referred to as supersaturation). In contrast, solubilization, e.g. by surfactants, does not lead to an increased concentration of molecularly dissolved drug.

In current literature, different terminologies are used for solubility, supersaturation, and solubilization of API in solutions. For this review, we propose to use the terminology shown in Figure 2, based on the work of Taylor & Zhang (2016). Solubility (of a dissolved API) generally refers to molecularly dissolved molecules of an API in an (aqueous) solution. However, two different states of the solution are possible: (1) solutions with a maximum concentration of the crystalline solubility and (2) supersaturated solutions with the maximum concentration of the amorphous solubility. Crystalline solubility (or just solubility) is a result of the thermodynamic equilibrium between an excess of crystalline and dissolved API in a dissolution medium, whereby strictly seen the crystalline structure should be the most stable polymorph. Dissolution from amorphous solids follows the same concept, except that this equilibrium is metastable, i.e. is not a thermodynamic equilibrium, and it exists between the amorphous state of the drug and its solution in the absence of any crystalline material. If a supersaturated drug solution exceeds the amorphous solubility, this amorphous phase will form spontaneously. This phenomenon is also referred to as liquid–liquid phase separation (LLPS) or glass–liquid phase separation (GLPS), depending on the glass transition temperature of the amorphous phase compared to the experimental temperature (for drug delivery mostly body temperature). As most authors do not distinguish between those two cases, we use the term amorphous-liquid phase separation (ALPS) as a combination of those two phenomena. In literature, the amorphous phase is also referred to as the drug-rich phase or drug-rich particles. In this review article, we reserve these terms for particles resulting from ALPS. This state of ALPS is thermodynamically metastable and crystallization of the drug will occur eventually.

Comparing crystalline solubility and amorphous solubility, supersaturation (also referred to as true supersaturation in literature) is the effect of the dissolution of more API than it would be possible taking the crystalline equilibrium solubility as a reference. Every concentration between crystalline and amorphous solubility can therefore be referred to as supersaturated. In contrast, solubilization refers to solubilizing API in solution by the help of additional excipients to form micelles or complexes (Taylor & Zhang, 2016). Solutions from ASDs often are mixtures of these states, which makes their analysis more complicated (Kanzer et al., 2010; Frank et al., 2012; Ueda et al., 2019). Therefore, terminology found in literature is the apparent solubility, which we use in this article as the maximum amount of detectable API in solution. Besides the molecularly dispersed API, this apparent solubility may include colloidal states of dissolved ASDs, such as drug-rich particles or micelles. In the literature, the exact definition of apparent solubility is often undefined and strongly depends on measurement methods. Similar problems were observed with the characterization of colloids evolving upon dissolution of ASDs. Often, it is unclear if drug-carrying colloids were formed or not, and what their physicochemical properties are. Where necessary, we interpreted the descriptions from authors and described the colloids according to the proposed nomenclature in this review. Despite the frequently undifferentiated description of solubilities and formed colloids, the identification of the underlying physicochemical properties is essential to advance the mechanistic understanding of drug uptake from solid ASDs.

## 5. Dissolution of ASDs

In the cascade for bioavailability, dissolution, i.e. formation of the dissolved ASD states is a first critical step. Even though this step cannot be completely delinked from later stages toward the uptake of API, in this review, we would like to commit a separate chapter to the dissolution of solids ASDs to dissolved ASDs.

As it is reported in the literature, ASDs can suffer from scarce or incomplete dissolution. This was shown, for example, in a study on ASDs of phenytoin and probucol as model drugs using different polymers (Dalsin et al., 2014). Depending on the polymer and drug load, the absence of dissolution and formation of colloidal states was observed. In addition, as also pointed out by Aleandri et al. (2018), the second dissolution step, where the API should be released from the colloidal states into the molecularly dissolved state, should be sufficiently effective in order to enable for absorptive flux across the intestinal epithelium. Further details on the absorption of drug from ASD solutions will be provided in Section 7.

### 5.1. Dissolution mechanisms

Craig (2002) established the concepts of carrier and drug-controlled release in ASD based on considerations of Simonelli et al. (1969). If the polymer does not dissolve into the dissolution medium, i.e. forms a highly viscous gel layer where the diffusion of an API molecule is slow as compared to pure solvent, the limiting step of release is the carrier. If the polymer dissolves into the dissolution medium, i.e. without a gel layer and drug particles are exposed to the dissolution medium, the dissolution process is drug controlled. These two rate-controlling processes can also co-occur (Vo et al., 2013). Even though this concept of the drug release assumes that the drug is heterogeneously dispersed in the carrier matrix, this approach still could be considered to be applicable even for homogenous dispersions. On the one hand, crystallization in the absence of the polymer could occur, i.e. when the polymer dissolves faster than the drug. On the other hand, the dissolution of crystalline drug could also be facilitated by the viscous gel layer (Punčochová et al., 2015; Szafraniec et al., 2018).

An alternative dissolution concept was proposed by Sun & Lee (2015). They compared medium-soluble and medium-insoluble carriers with indomethacin as a model drug compound. Authors distinguished according to the solubility of the carrier in the release medium between (1) dissolution-controlled release (for medium-soluble carriers) and (2) diffusion-controlled release (for medium-insoluble carriers). In the first case, the carrier is quickly transiting into dissolved or colloidal states, thus supersaturation can be achieved due to the fast liberation and subsequent dissolution of the amorphous API. Thereby, the dissolved polymers inhibit crystallization of the supersaturated solution, which would recrystallize fast due to the fast supersaturation rate (also compare to Section 6.2.1) (Sun & Lee, 2013). In contrast, in the second case, the release is based on a continuous diffusion of the API from the carrier matrix into the release medium, which can be interpreted as a carrier-controlled release process. The driving force of this process is the gradient of the drug concentration between the carrier and the release medium. As a consequence, the API concentration in the release medium will not exceed the API concentration in the ASD. In case of a reduction of the API concentration in the release medium, more drug will diffuse from the drug carrier into the release medium. In other words, the carrier serves as a depot, regulating the maximum possible drug concentration in the release medium. This concept is especially important in the light of another research work (also refer to Section 6.2.1) (Han & Lee, 2017), proposing that crystallization is not induced when drug concentrations in the dissolution medium are under a critical concentration. These reports are in line with a review on polymeric ASDs formulations by Baghel et al., where the dissolution of ASDs is split into two scenarios: rapid dissolution and subsequent crystallization from the solution of increased apparent solubility or slow dissolution and crystallization of the API from the ASDs during dissolution (Baghel et al., 2016).

Sun and Lee do not report the formation of a drug-rich phase in the dissolution-controlled case. However, at least for certain formulations, the formation of colloidal states is possible for this dissolution scenario, as reported by Saboo et al. (2019): the congruent release of polymer and API (dissolution controlled release) was proposed to be essential for the formation of particles by ALPS, which is in line with the concept of the dissolution controlled release.

The formation of drug-rich particles from carrier-controlled release was proven in a study by Indulkar et al. (2017). They investigated the origin of drug-rich particles by the example of ASDs of nifedipine with HPMC (hydroxypropyl methylcellulose) or PVP-VA (polyvinylpyrrolidone-vinyl acetate) using isotope scrambling in combination with NMR (nuclear magnetic resonance) spectroscopy. Authors distinguished two theoretical principles of the formation of drug-rich particles: (1) the molecular dissolution of the API and subsequent phase separation if the concentration exceeds the amorphous solubility (carrier-controlled dissolution) and (2) the dispersion of drug-rich domains already existing in the solid ASD. For the ASD investigated, authors experimentally proved the first mechanism to be applicable.

A dissolution concept based on imaging experiments was proposed by Punčochová et al. (2015). The authors investigated ASD dissolution mechanisms using ATR-FTIR (attenuated total reflection Fourier transform infrared) and NMR imaging on the example of three polymers and aprepitant as a drug substance. They identified the following release process from polymer matrix: water ingresses into the tablet, the polymer begins to swell, the drug diffuses out of the swollen matrix and polymer starts to erode. They also found that the gel layer can stabilize the API in the supersaturated state. Consequently, fast polymer erosion can lead to drug crystallization. The API dissolution, in this case, is controlled by the diffusion through the gel layer (carrier-controlled dissolution) and in the early stage of dissolution also by the water ingress (if no disintegrant was used). This is in line with a study conducted by Dahlberg et al. (2010b). They showed that water ingress rate has no direct influence on the release kinetics and drug release correlates with polymer mobilization kinetics.

**Figure 3.**
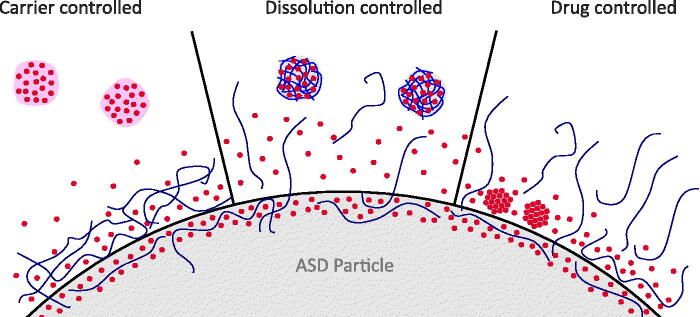
Three main concepts for dissolution from ASDs. (1) In the case of carrier controlled release, drug molecules have to diffuse through the polymer, possibly through a highly viscous gel layer on the surface of ASD particles. If dissolved drug concentrations become high enough to exceed the amorphous solubility, ALPS will occur, inducing the formation of drug-rich particles. (2) In the case of dissolution controlled release, API and polymer dissolve congruently, leading to fast dissolution and formation of drug-rich particles. The polymer may stabilize the supersaturated solution. (3) In the case of drug controlled release, the polymer dissolves out of the ASD and the residual API controls the dissolution rate. If the residual API is not stable in the amorphous state without polymer, i.e. crystallizes, supersaturation will not occur.

In summary, there is accumulated evidence in the literature, that mainly three mechanisms of the dissolution of ASDs occur (Figure 3):

1. *Carrier controlled release*. Water ingresses into the polymer and induces the formation of a highly viscous gel layer, through which the API has to diffuse. This usually results in a slower release, where the concentration in the dissolution medium is controlled by the API concentration in ASDs and the volume of the release medium. If amorphous solubility is exceeded, drug-rich particles can form.
2. *Dissolution controlled release (congruent release)*. API and polymer release simultaneously and fast into the dissolution medium, leading to significant supersaturation effect. Here, the polymer in solution is essential to stabilize the supersaturated state. The supersaturation concentration is controlled by the total drug dose and the volume of the release medium.
3. *Drug controlled release*. The polymer dissolves into the dissolution medium and the remaining amorphous API dissolves at a drug-controlled rate. For this mechanism, there is a risk of crystallization of the API already during the dissolution process. In theory, also here drug-rich particles could form if the amorphous drug state is stable enough, but experimental data were not found for this review.

Besides feasibility considerations for the three different dissolution approaches for a specific API, it remains up to the formulation scientist to choose between these options based on further considerations such as the desired onset of action of the drug.

### 5.2. Factors affecting dissolution

Based on the abovementioned mechanistic considerations, factors that affect ASD dissolution may be deduced. Besides focusing solely on mechanistic understanding of dissolution of ASDs, different authors investigated factors that affect the dissolution of ASDs from solid to a dissolved state. This section reviews such factors.

#### 5.2.1. Effects of drug load

It seems well established, that drug load has a direct impact on dissolution and subsequently on the properties of the dissolved ASDs. This has been shown, for example, in a study on ketoprofen by Manna et al. (2007). In more detail, Tres et al. (2018) showed that higher drug loads can reduce the release rate and affect the final state of the dissolved ASDs. In their article, higher drug loads (50%) compared to lower drug loads (10%) of ritonavir in PVP-VA showed a dissolution pattern of a drug-controlled release for the hydrophobic APIs. The resulting drug concentration in the dissolved state of ASDs was below the amorphous solubility. In another study, Tres et al. (2014) used real-time Raman imaging to investigate dissolution mechanisms of ASDs tablets by the example of felodipine in PVP-VA at two drug loading levels. At the low drug load level (5%), a congruent release of drug and polymer was observed, showing no indication of crystallization. At the higher drug load level (50%) however, drug crystallization was observed after a loss of polymer from the ASD, resulting in slow dissolution. A heterogeneous crystallization process proposed based on the observation of different rates of phase transition at different locations of the dosage form.

In conclusion, even though higher drug loads are desired in formulation development for a low pill burden, negative effects of high drug load have to be accounted for. It can be hypothesized that higher drug loads prevent dissolution controlled (congruent) release and therefore induce drug-controlled release, failing to achieve the desired effect of supersaturation.

#### 5.2.2. Effects of the degree of ASD homogeneity

Molecular arrangements within ASDs play an important role as has been shown by various research works. A study on the emerging of colloids from probucol in HPMC formulated together with sodium lauryl sulfate (SLS) showed that a completely amorphous state of a material is important for an efficient dissolution (Zhao et al., 2019). The presence of crystalline domains in the ASD changed the behavior of particles emerging from ASDs. Completely amorphous ASD generated colloids containing no crystal phase. In contrast, colloids evolving from ASDs with residual crystallinity or amorphous drug domains in the solid state, showed phase separation within the colloidal particles and subsequent growth of large crystals (all observations shortly after ASD dissolution). These findings are in line with a recent study by Wang et al. (2018) where the quality of the ASD, namely the degree of homogeneity had a direct impact on dissolution results. As shown for posaconazole in HPMC-AS (hydroxypropyl methylcellulose acetate succinate), a homogenous distribution of the drug in the polymer matrix led to the simultaneous release of drug and polymer (dissolution-controlled release). A non-homogeneous distribution led to a faster release of the polymer than the drug (drug-controlled release). Also, Baghel et al. (2018) pointed out that intimate drug–polymer mixing is of great importance to increase the apparent solubility upon ASD dissolution.

Therefore, an incomplete amorphization and homogenization might be a pitfall for efficient dissolution and subsequent stabilization of the resulting colloids, setting high requirements to the production process as well as the analytics of the solid ASDs.

#### 5.2.3. Effects of wetting properties

Before any dissolution, wetting of the ASD particles is essential and therefore has a direct impact on the dissolution kinetics: a study investigating the effect of wetting kinetics of ASDs showed that, as expected, faster wetting kinetics correlated with faster dissolution rates (Verma & Rudraraju, 2015). However, ASDs did not always show the expected behavior based on their composition. In contrast to physical mixtures of API and polymer, the wetting behavior of ASDs in some cases could not be explained by the wetting behavior of the individual components, as shown by Dahlberg et al. (2010a) by a set of ASD formulations with HPMC as polymer: the surface was always more hydrophilic than any of the individual compounds. They propose that the molecular interaction of API and polymer leads to a rearrangement of the polymer, orienting hydrophilic groups toward the surface.

#### 5.2.4. Effects of drug–polymer interactions

In the literature, also the impact of drug–polymer interactions on the dissolution performance was investigated. A study by Chen et al. (2016a) investigated the formulation factors that control drug and polymer dissolution rates by an example of ketoconazole ASDs of different polymers. Authors distinguished the systems’ behavior according to the drug–polymer interaction, represented by Flory–Huggins interaction parameter. For systems with considerable interactions and homogenous ASD mixing, a congruent drug and polymer release was observed (dissolution-controlled release). In contrast, slow drug release rates independent of the polymer release rate (drug-controlled release) were observed for systems with poor polymer–drug interactions as well as for ASDs with considerable interactions but inhomogeneous mixing. These findings stand in contrast to the article by Kaushal et al. (2004), describing that strong drug–carrier interactions decrease the release rate. From a conceptual point of view, latter could be expected for carrier-controlled release, where strong interactions might prevent efficient drug diffusion.

Scientifically, it would be interesting to investigate the relation between drug–polymer interactions and dissolution properties in more detail, as it elucidates molecular mechanism of drug dissolution of ASDs. In formulation development, this would be especially interesting because these interactions can also be modeled or calculated, which could allow for early predictions.

#### 5.2.5. Effect of surfactants

Also, in classical formulations, surfactants have been used frequently to affect the dissolution performance. Similarly, when embedded into ASDs, surfactants have been used to enhance dissolution rates, even though also negative effects of surfactants have been reported (Ghebremeskel et al., 2007).

A detailed study of Meng et al. (2019) on celecoxib ASDs based on PVP and TPGS (tocopheryl polyethylene glycol succinate) as surfactant showed that surfactants impact the ASD dissolution behavior in different ways at a threshold of 20% TPGS in the ASD: improved powder wetting, enhanced formation of nano-aggregates (drug-rich particles), and a solubilization by TPGS micelles. Celecoxib was also found to improve miscibility between TPGS and PVP. At the highest level of TPGS contents, colloids were significantly larger, which, based on the known size of TPGS micelles, should not to be considered micelles. It could be concluded, that TPGS promoted a formation of drug-rich particles. By the example of itraconazole ASDs with HPMC-AS (Solanki et al., 2019), it was shown that the addition of surfactants (Poloxamer 188, Poloxamer 407, and TPGS) enhanced dissolution and supersaturation effects. Furthermore, authors observed the formation of fine particles (drug-rich particles) in the dissolution medium.

While surfactants seem to be able to enhance ASD dissolution properties, it is essential to also investigate their effect on the formation of colloids, which subsequently has a direct impact on drug uptake (refer to Section 7).

#### 5.2.6. External factors

Dissolution is also influenced by external factors, for example, dissolution media. Luo et al. (2019) proposed, based on experiments on a tanshinone IIA in a chitosan system, that even for a single polymer, different dissolution mechanisms can be relevant based on the pH of the dissolution medium. At a low pH, polymer degradation seems to be relevant, while at a higher pH swelling was observed. Furthermore, especially in the presence of surfactants, ionic strength also has a direct impact on dissolution performance (Fong et al., 2016).

As summarized by Fotaki et al. (2014), the use of appropriate media in dissolution tests is of high importance during ASD development. Especially, it concerns application of biorelevant media (SGF, FaSSIF, and FeSSIF), as it has a crucial impact on ASDs’ dissolution behavior, i.e. dissolution rate, supersaturation degree, and crystallization. Investigation of dissolution behavior in biorelevant media is indispensable for prediction of *in vivo* results. While more investigations on the effect of dissolution media and their relevance in ASD development are necessary, the combination of experiments in different media might be the most promising approach to discriminate between different ASD formulations as well as to provide insights into ASD behavior under physiological conditions.

## 6. Dissolved ASDs: a complex mixture

Dissolved forms of ASDs may exist in many forms and morphologies as will be shown in this section. The behavior of the different forms is regulated by different physicochemical mechanisms. In literature, the dissolved form of ASDs is often insufficiently characterized, e.g. it is often not indicated if colloidal states form and if yes, what kind. It therefore can be assumed that the formation of colloidal states is underreported in literature.

**Figure 4.**
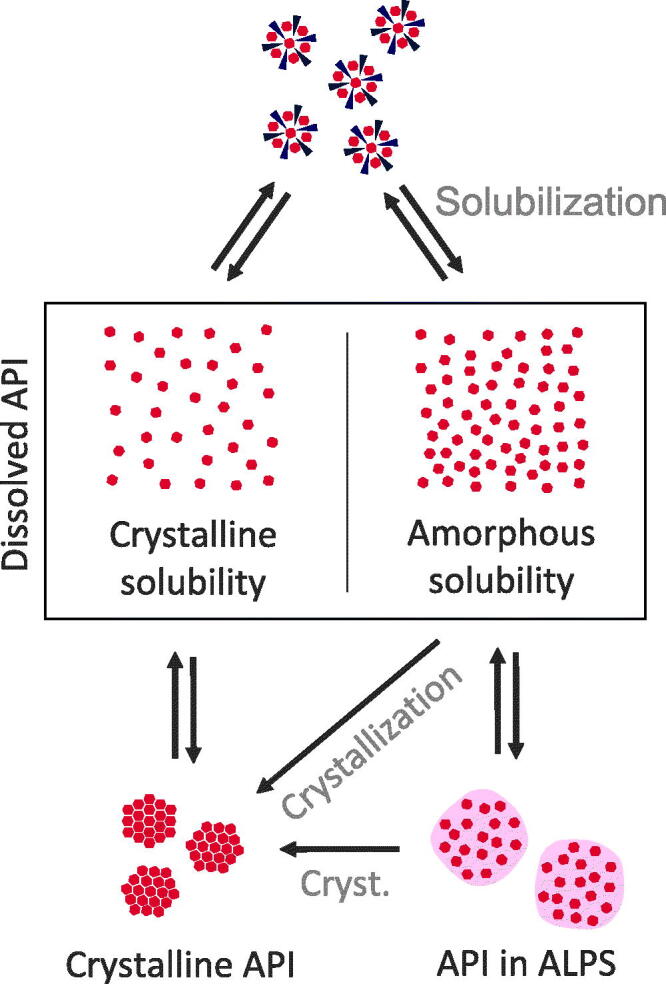
Classifications of states within dissolved ASDs. Dissolved API is in equilibrium with crystalline API or API in ALPS. The supersaturated state and, if the amorphous solubility is exceeded, ALPS are in a metastable state, where crystallization can occur (cryst.). Crystallization will lead to a reduction in concentration of the dissolved state down to crystalline solubility. Furthermore, there is an equilibrium between solubilized API and both dissolved states of dissolved API, i.e. the supersaturated and the not supersaturated state.

Often, a complex mixture of colloidal states emerges upon dissolution. An example of the complexity of the dissolved forms of ASDs was investigated by Frank et al. (2012). Using AFlFFF (asymmetrical flow field-flow fractioning), they investigated the composition of different colloidal states evolving from ASDs of ABT-102 (a low soluble small molecule compound), PVP-VA, and surfactants. Authors described different fractions, namely polymeric, micellar, and microparticulate ones. The microparticles were found to consist mainly of amorphous API with surfactants, the latter being attributed to a stabilizing effect on the amorphous particles. Even though the drug was mostly found to be incorporated in the colloids, the molecularly dissolved drug concentration was also found to be increased compared to the crystalline solubility, most likely due to ALPS. Similar observations were made by Kanzer et al. (2010), who identified comparable species (namely colloidal polymer, drug rich nanoparticles, and nanoparticulate assemblies of sorbitan monolaurate, and hydrophilic fumed silica) by the examples of lopinavir and ritonavir. A direct influence of the ASD composition on the type of colloidal states was also described in the literature (differentiated by amorphous droplets, amorphous particles, and gel-like particles) (Ueda et al., 2019). Figure 4 summarizes states of dissolved ASDs and their interactions.

### 6.1. States of dissolved ASDs

#### 6.1.1. Dissolved API

As pointed out in Section 4, molecularly dissolved API can either exist in a thermodynamically stable equilibrium with crystalline API or in a supersaturated state in a metastable equilibrium with amorphous API. The stable state is limited by the API solubility, which is poor by default for BCS (biopharmaceutics classification system) class II and IV drugs. If the API is present in amorphous form, supersaturation in a molecularly dissolved state can be established. This state of supersaturation is only stable until the first crystalline phase is formed (Taylor & Zhang, 2016).

#### 6.1.2. Solubilized API

Colloidal systems dissolving from ASDs can form based on contributions from the ASD polymers, additional excipients such as surfactants, APIs themselves, or combinations thereof. The formation of micelles from surfactants is described elsewhere (Maibaum et al., 2004; Cui et al., 2008; Xu et al., 2013b) and therefore not discussed further in this review article. One particular case is Soluplus (polyvinyl caprolactam–polyvinyl acetate–polyethylene glycol graft copolymer), which is not only a suitable base polymer for ASD formulation by hot-melt extrusion but also a polymer that forms micelles upon dissolution, as was shown by Zi et al. (2019). Another study (Rani et al., 2019) showed that polymeric micelles evolving from a ASD composed of curcumin and Soluplus form hydrophobic cores within the polymeric micelles. These cores showed higher rigidity than the outer layers based on fluorescence anisotropy and micropolarity measurements. This core rigidity phenomenon is explained by the formation of multiple h-bond between curcumin and Soluplus, as shown by infrared spectroscopy. This work indicates that besides the pure physicochemical aspects of micelle formation, specific excipient–API interactions have a significant effect on the formulation behavior.

#### 6.1.3. API in amorphous-liquid phase separation

In literature, different examples of GLPS (Mosquera-Giraldo & Taylor, 2015; Mosquera-Giraldo et al., 2018b), and LLPS (Ilevbare & Taylor, 2013; Indulkar et al., 2016; Saboo et al., 2019) (together referred to as ALPS in this review) evolving during ASD dissolution were reported. As already pointed out in Section 4, from a physicochemical point of view, ALPS only occurs if supersaturated drug concentration exceeds the amorphous solubility (Taylor & Zhang, 2016; Baghel et al., 2018; Stewart et al., 2019). However, beyond the formation of ALPS based on phase separation from pure API-solvent mixtures, polymers play an important role in the formation of ALPS-particles. For example, systems of nifedipine and HPMC derivatives (HPMC acetates and succinates), showed in NMR experiments that the more hydrophobic the polymer (depending on polymer derivative and pH), the higher is the distribution of the polymer into the ALPS-particles. In these particles, polymers were able to inhibit crystallization (Ueda et al., 2017). Similar results were demonstrated in a study of a posaconazole–HPMC–AS system (Chen et al., 2016b).

#### 6.1.4. Crystalline APIs

Even though drug delivery by ASDs aims to deliver API in an amorphous form, the possibility of remaining crystallinity should not be overlooked. A study (Szafraniec et al., 2018) on bicalutamide using Poloxamer 407 and PEG (polyethylene glycol) 6000 showed an enhanced *in vitro* performance despite detectable crystalline API in the formulation. A size reduction of the crystalline particles in the polymer matrix, without or with an incomplete ASD formation, seemed not to have an influence on the ASDs performance as well as on the formation of Poloxamer particles. Authors concluded that an enhanced wetting and the solubilization of the API enclosed into nanoaggregates of Poloxamer 407 were the basis for enhanced dissolution properties. This study shows that amorphization could not be the only mechanism toward increased bioavailability through ASDs, but that also locally increased solubility of the crystalline API could be beneficial.

### 6.2. Stabilization of dissolved ASDs

While the existence of supersaturation and colloidal states is possible to study experimentally, mechanisms involved to stabilize these metastable systems are more complex to investigate. From a drug delivery perspective, this metastable, supersaturated state is the key for enhanced bioavailability. Therefore, the state of supersaturation should be stable for a period long enough for the API to be absorbed (Baghel et al., 2016). While a phase separation from the metastable system is frequently observed, the nature of those precipitates (e.g. their solid-state or composition) is often not investigated in detail (Khan et al., 2016).

#### 6.2.1. API-dependent supersaturation stability

Drug crystallization from supersaturated solutions and corresponding kinetics are governed by complex mechanisms. It can be assumed that the stability of the supersaturation of the pure drug in solution is important also in the presence of polymers, which can stabilize the system additionally. It was proposed by Baghel et al. that mechanisms of crystallization inhibition with and without excipients interlink at the step from the supersaturation generation phase to the supersaturation stabilization phase (Baghel et al., 2018).

A study by Raina et al. (2015a) investigated the effects of polymers on crystallization from supersaturated solutions by using wide-angle X-ray scattering synchrotron radiation. It was shown that the crystallization times of the pure drug solutions of six dihydropyridine calcium channel blockers were very heterogenous, allowing for classification into slow or fast crystallizers. This is in line with a study analyzing the crystallization behavior from a dissolved amorphous form of approximately 51 different drugs showing that the time to crystallization varies significantly between different drugs, ranging from immediate and complete crystallization to absence of any observable crystallization (Van Eerdenbrugh et al., 2014). Authors mention that previous studies showed that lower molecular weight and less complex structures and fewer rotatable bonds in a chemical structure of the drug substance are indicators for rapid crystallizers (Baird et al., 2010; Van Eerdenbrugh et al., 2010). This is in line with a report showing that a higher molecular weight, together with a low level of symmetry and small electronegative atoms (are indicators that such drug molecules can form glasses, Mahlin et al., 2011).

A study by Sun & Lee (2013) showed that the crystallization rate is strongly dependent on the supersaturation rate in pure drug solutions. The faster the supersaturation, the faster the subsequent crystallization. Authors also provide a mechanistic model based on crystal nucleation and growth, allowing for prediction of the optimal supersaturation rate for a maximized area under the dissolution curve. They also showed that the crystallization rate has a direct influence on which drug polymorph will be formed during crystallization.

A study proposing the concept of optimal AUCs was published by Han & Lee (2017). Authors describe that the supersaturation effect is less useful at maximal supersaturation (due to fast recrystallization), but beneficial if the concentrations are kept below the critical supersaturation concentration *S*_c_. They showed that below this concentration, no detectable crystallization occurred during the time of observation and therefore supersaturation was maintained and experimentally proved the existence of *S*_c_. Results were in good agreement with a mechanistic model based on the classical nucleation theory. In solutions with polymers, they observed similar trends, however, *S*_c_ was higher due to crystallization inhibition by the polymer (indicating additive effects of polymers). As the dose is directly related to the maximum concentration and the dissolution rate in constant volume, they propose that there exists an optimal dose that will maximize the area under the curve (AUC) for the dissolution profile.

In summary, based on considerations for pure drug solutions, the stability of the supersaturated state is highly dependent on the drug itself. More complex drug molecules seem more likely to be stable in the supersaturated state. A fast supersaturation can also trigger fast crystallization, which is why a controlled supersaturation rate might be desirable. Where reasonable, concentrations below the critical supersaturation concentrations should be chosen for maximum stability.

#### 6.2.2. Supersaturation stabilization by polymers

In this section, we review mechanistic insights of crystallization inhibition of supersaturated solutions by polymers. Their effect varies significantly, as shown in a study by Curatolo et al. with 41 potential precipitation inhibitors (including polymers and other substances) to stabilize nine different APIs in a supersaturated state (Curatolo et al., 2009). Some underlying principles of supersaturation stabilization by polymers have already been investigated independently of ASDs, which reasoned earlier reviews on this topic by Warren et al. (2010) as well as Xu & Dai (2013a), where the effect of precipitation inhibition based on physicochemical interactions is described. In brief, authors identified multiple mechanisms leading to crystallization inhibition by polymers:

- Changes in solution properties (increasing solubility and viscosity);
- Changes in the adsorption layer in the crystal (decreasing the diffusion through the layer);
- Changes of the crystal surface (polymer adsorption on crystal and steric hindrance of crystal growth, smoothing of imperfections and therefore eliminating growth spots, altering the surface energy);
- Molecular interactions (hydrogen bonding, hydrophobic interactions with the best stabilization effect at an intermediate polymer hydrophobicity, higher polymer rigidity and molecular weight).

In this article, we would like to complement these reviews by several recent contributions to the field, with respect to delivery through ASDs.

Several experimental studies highlighted the role of molecular interactions to inhibit crystallization. In a work by Kojima et al. (2012), ASDs from mefenamic acid in Eudragit EPO (butyl methacrylate-co-(2-demethylaminoethyl) methacrylate-co-methyl methacrylate) showed that upon dissolution, a significant increase in solubility was achieved along with solution stabilization. Different molecular interaction between drug and polymer (ionic, hydrogen bonds, or hydrophobic), especially between the carboxyl group in mefenamic acid and the aminoalkyl groups in Eudragit EPO, were observed. Authors conclude that supersaturation is most likely facilitated by these interactions. Furthermore, the authors reported enhanced dissolution profiles for Eudragit EPO with the NSAIDs (non-steroidal anti-inflammatory drugs) indomethacin and piroxicam. There are also indications that a nonspecific binding can result in a crystallization inhibition effect (Baghel et al., 2018): an NMR study on nimodipine with PVP (polyvinylpyrrolidone) as a polymer in solution showed nonspecific hydrophobic interactions between the hydrophobic moieties of the polymer and the drug (Pui et al., 2018). A study investigating the higher degree of crystallization inhibition by PAA (polyacrylic acid) compared to PVP concluded that this effect is attributed to strong specific interactions between drug and polymer as observed with NMR measurements. In a study on supersaturation stabilization of hydrocortisone acetate by HPMC authors (Raghavan et al., 2001) hypothesize that the adsorption of polymer on the crystals facilitated by hydrogen bonding is the mechanisms behind crystallization inhibition.

Several research groups have investigated the effects of structural properties of polymers on crystallization inhibition. Mosquera-Giraldo et al. (2018a) looked at nine drugs (ritonavir, nifedipine, celecoxib, atazanavir, nevirapine, ezetimibe, telaprevir, griseofulvin, and danazol) in the presence of five different synthesized cellulose derivatives (shorter, longer, branched, or unbranched side chains with carboxylic acid or alcohol termination) with respect to the crystallization inhibition capacities. Most effective in the prevention of drug crystallization were polymers with a short side chain and one carboxylic terminal group, while polymers with a longer side chain and two carboxylic terminal groups were less effective. Molecular dynamics simulations with one polymer chain in the presence of drug and water molecules showed that for more effective polymers, there was a higher probability of interactions between drug and polymers as well as more negative values for estimated free energies of interaction. The study shows that molecular dynamics simulations are a useful tool for the prediction of crystallization inhibition. The analysis of effects of polymers on crystallization in a study of Khan et al. (2016) revealed that polymers with intermediate hydrophilicity/hydrophobicity were most effective in delaying crystallization while strongly hydrophilic or strictly hydrophobic polymers did not show such an effect. Based on NMR spectroscopy results, authors conclude that the interaction of polymers with the hydrophobic drug-rich phase and the aqueous phase is a prerequisite for stabilization of supersaturation by polymers. These results are supported by a study correlating the potential to inhibit the crystallization of ritonavir of 34 polymers based on their physical and chemical properties (Ilevbare et al., 2012b). Authors showed that polymers with a moderate level of hydrophobicity, semi-rigidity, and a high amphiphilicity (many ionizable groups) inhibited crystal growth most successfully. This effect was most expressed by novel cellulose derivatives. Authors attributed these properties to be important for polymer absorbance on ritonavir crystals. This was also reported in the already mentioned study of Curatolo et al. (41 precipitation inhibitors tested on nine drugs), where HPMC-AS exhibited best *in vitro* stabilization performance (Mahlin et al., 2011).

ASDs can also be formulated as a combination of excipients (additional of surfactants alone are discussed in the next section). A study analyzing the effect of polymers, surfactants, and their combinations on crystallization inhibition reports mostly synergistic effects of polymer–polymer combinations (Ilevbare et al., 2012a), especially for the combination of two moderately hydrophobic polymers or a moderately hydrophobic polymer with a more hydrophilic polymer. These effects were attributed to polymer–polymer hydrophobic interactions, enhancing interaction with crystallizing solutes and growing crystals. In contrast to polymers, the addition of surfactants mainly negated the positive effects of the polymers, which was attributed to the hindrance of interactions of polymer and API by the surfactants.

Looking at the kinetics of crystallization, a study on the crystallization of felodipine in the presence of HPMCP (hydroxypropyl methylcellulose phthalate) showed that delaying nucleation has a stronger kinetic effect compared to the reduction of the crystal growth rate. Authors therefore conclude that the absence of residual crystallization centers in the ASDs is of crucial importance (Alonzo et al., 2012).

From a drug delivery perspective, not only the complete prevention of crystallization can be beneficial. A study by the example of nifedipine (Raina et al., 2014) showed that that polymers in solution might not be able to prevent crystallization but have an influence on the polymorph formation (as discussed for pure API solutions, Sun & Lee, 2013). If this polymorph has an increased solubility, bioavailability could be increased.

In summary, various studies have shown that crystallization inhibition by polymers is possible. Underlying mechanisms were often traced back to molecular interactions (ionic, hydrogen bonding, and hydrophobic) of the polymer with the drug in solution or the drug-rich phase. Favorable physicochemical properties of polymers were reported to be intermediate hydrophobicity as well as high amphiphilicity with sufficient ionizable groups. Furthermore, hydrogen bond donors and acceptors and sufficient molecular weight are favorable. Different polymers can also have an additive effect on crystallization inhibition. It seems evident that during polymer selection as well as for their fraction in the formulation (Verma & Rudraraju, 2015), scientists should not only bear in mind requirements to the solid state of the ASD (the formation and stability), but also for supersaturation stabilization (Baghel et al., 2018). Based on the advantage of molecular interactions for the stability of both, the solid and dissolved state, a possible correlation of amorphous stabilization in solid and dissolved state was also proposed in the literature (Chauhan et al., 2013).

#### 6.2.3. Effects of surfactants on crystallization inhibition

Surfactants are frequent additions to polymeric ASDs. Different authors reported positive as well as negative effects of surfactants with respect to crystallization inhibition. An example is a report by Mosquera-Giraldo et al., where surfactants (SLS, sucrose palmitate, and TPGS) increase the crystallization rate of supersaturated celecoxib. While the presence of the PVP could again decrease crystallization rates, the effect of the surfactants was still observable (Mosquera-Giraldo et al., 2014). In another study, inhibition of crystallization in drug-rich particles by surfactants was reported (Frank et al., 2012). Similar observations were reported by Chen et al. for sodium taurocholate, which was significantly prolonging times to nucleation for 11 diverse drug substances. Therefore, also endogenous substances, such as bile salts, might stabilize drug molecules in their amorphous solid form (Chen et al., 2015a). These controversial findings are in line with a report (Chen et al., 2015b) by the example of celecoxib ASDs of HPMC-AS on the impact of surfactant on the nucleation times in aqueous suspensions. Authors showed that some surfactants like SLS and polysorbate 80 promote crystallization, whereas other substances such as sodium taurocholate or Triton X100 inhibit crystallization. In addition, authors found a destabilizing effect through the increase in the dissolution rate of the drug.

Also, investigations of underlying mechanisms for the effects of surfactants were published. It was reported by the example of a posaconazole HPMC-AS ASD that SLS in the solution destabilized the drug-rich phase (based on ALPS) and led to an early crystallization. The polymer alone had a stabilizing action on the drug-rich phase compared to the drug alone. Authors showed that SLS competitively interacts with HPMC-AS, therefore reducing its stabilization action for posaconazole. The negative effect of SLS was confirmed in a cross-over PK (pharmacokinetic) study in dogs, where the formulation with SLS only showed 30% of the bioavailability compared to the formulation without SLS (Ueda et al., 2017). A study on nimodipine with PVP as polymer in solution by NMR spectroscopy showed nonspecific hydrophobic interactions between the hydrophobic moieties of the polymer and of the drug (Kojima et al., 2012). The addition of SLS showed induction of crystallization at a lower concentration, while higher concentrations were slightly increasing the supersaturation, however, only in combination with PVP. In contrast, low concentrations of sodium taurocholate increased the supersaturation, while the higher concentrations slightly induced crystallization. Authors suggest a competitive binding of a surfactant to the polymer as well as a capacity of surfactants to interact with drug molecules.

As it was also pointed out by Chaudhari & Dugar (2017), besides of effects of surfactants below their critical micelle concentration (CMC), surfactants can also inhibit API precipitation above their CMC, i.e. through API partitioning into micelles. This partitioning can reduce the fraction of molecularly dissolved API, known as a true supersaturation, and therefore thermodynamically stabilize the drug solution (Feng et al., 2018). Furthermore, a more detailed systematic study on the effects of surfactants on API stabilization below and above of the CMC was conducted by Zhang et al. (2019). The authors observed varied effects from surfactants on de-supersaturation, both promoting or reducing at surfactant concentrations below or above CMC, respectively. They conclude that these effects seem to be API specific and that currently a screening approach is the only way for formulation development. In the context of this review article, stabilizing systems with surfactant concentrations above CMC are not classified as molecularly dissolved API, i.e. formation of a true supersaturation phase. The formation of micelles influences drug uptake, as discussed in Sections 7 and 8.

Even if the complete mechanisms of these complex interactions are not known yet, it seems evident that a side from positive effects, surfactants can also have negative effects on the stability of supersaturated drug solutions. More research to elucidate more detailed mechanisms of how surfactants affect the stability of supersaturated API-solutions will be necessary to finally estimate if the addition of a certain surfactant will be beneficial or not.

## 7. Uptake from dissolved ASDs

### 7.1. Mechanisms of uptake from dissolved ASDs

A fundamental question of uptake from dissolved ASDs is to identify if molecularly dispersed API is the only fraction that is taken up or if entire particles can be absorbed, e.g. by M-cells. A review (Buckley et al., 2013) looking at the uptake from enabling formulations for poorly soluble drugs concluded that based on experimental results from available studies, only supersaturated (i.e. molecularly dissolved) API can increase the transmembrane flux. In contrast, solubilized API (e.g. in micelles from endogenous bile salts or surfactants contained in the formulation) might limit the transport. This is corroborated by a study on biomimetic micelles, where authors conclude that even the uptake of particles mimicking endogenous structures is unlikely (Ma et al., 2017). Furthermore, active uptake mechanisms were not observed for ASDs: using single-pass intestinal backflow (in rats), Cheng et al. (2010) investigated the uptake of bifendate delivered in form of an ASD. There were no indications found that the transport mechanisms were active. Passive diffusion was also observed in a study on α-asarone, which showed an enhanced *in vivo* bioavailability through ASD formulation (Deng et al., 2017). Therefore, in this section, we assume that only molecularly dissolved API is absorbed significantly by the intestinal epithelium and that this uptake is generally passive (except for API-specific active transport). Alternative routes of uptake are discussed in the next section.

Different studies aimed to elucidate the correlation with the degree of (apparent) supersaturation and transmembrane flux. Shi et al. (2015) showed that the increase of the drug’s apparent solubility by micellization had little effects on the concentration of molecularly dissolved drug in the release medium or the permeation rate of berberine from an ASD with hydrogenated phosphatidylcholine. It was shown that only the higher concentration of molecularly dissolved drug (true supersaturation) was relevant for an increase in permeation rate. Furthermore, the presence of drug-rich particles in aqueous dispersions induced supersaturation stability and enhanced the permeation. These results are in line with a report of Fong et al. (2016), reporting the absence of any correlation of enhanced apparent solubility of celecoxib in a solid phospholipid dispersion and permeability. A further study by this research group (Jacobsen et al., 2019) concluded that only elevated concentrations of molecularly dispersed API could increase the permeability. Comparable results were reported by Ueda et al. (2014), where authors measured the impact of crystallization inhibition by bile acids and lipid micelles solutions on permeation of dexamethasone. Authors showed that the partitioning of API into the micellar phase decreased permeation.

ASDs are not the only known supersaturating (solubilizing) drug delivery systems. Different studies report the effects of different formulation strategies on drug uptake. A study by Miller et al. investigated the effect of increased apparent solubility of progesterone by micelle formation (Miller et al., 2011) (as well as cyclodextrins (Dahan et al., 2010) and cosolvents (Miller et al., 2012b)) on permeability. The model used in this study considered diffusion through an unstirred water layer as well as the membrane transport, resulting in combined permeability. For the case of micelles, the transfer through the unstirred layer increased with increasing surfactant concentration, especially for concentrations greater than CMC. In contrast, the permeability across the membrane decreased with increasing surfactant concentration, also mainly for concentrations exceeding the CMC, due to a reduced fraction of molecularly dissolved drug. Authors validated this model for progesterone in a rat jejunal perfusion model. The model suggests a tradeoff between increased apparent drug solubility and decreased permeability for optimal drug exposure. These findings are in line with a report by Stewart et al. showing that drug-rich particles and micelles increased the diffusion through the unstirred layer. They identified a direct relationship between the diffusion coefficient of the particles and their percentage of drug load and permeation. The contribution of drug-rich particles to the number of diffusing particles was strongest when unbound and micelle-bound drug concentrations were kept low, indicating a high fraction of the API residing in the drug-rich particles (Stewart et al., 2017). A special case among micelles is reported for Soluplus micelles, produced by dissolving hot-melt extrudate from Soluplus and poorly soluble compound cyclosporine A. Here, solubilization in micelles increased permeation flux and *in vivo* bioavailability, however, formulations with strongest supersaturation effects did not perform best (Yu et al., 2013).

Compared to other supersaturating (solubilizing) drug delivery systems, ASDs show advantages with respect to bioavailability. In a further study by Miller et al. (2012a), authors investigated the effect of particles emerging from ASDs (progesterone in HPMC-AS) by applying an adapted model and equivalent validation in the rat jejunal perfusion model as mentioned above. In contrast to solubilizing strategies, particles formed from ASDs did not reduce permeation through intestinal wall, which is a crucial advantage of ASDs over solubilizing formulations. Authors showed that there is a constant PAMPA (parallel artificial membrane permeability assay) and jejunal permeability for all measured apparent solubility values of the tested formulation, indicating the absence of hindrance in permeability through the ASD formulation. Accordingly, the transmembrane flux is directly proportional to drug concentration in the donor compartment. Authors do not indicate the formation of drug-rich particles nor ALPS in their publication. Based on the linear increase in flux, we assume that the applied concentration range was below the ALPS concentration since above the ALPS concentration the further increase in flux would not be expected (Raina et al., 2015b). This is supported by a study of Frank et al. (2014): on the example of ABT-102 in a hydrophobic polymer and three surfactants, they investigated mechanisms of increased permeability through micelles and particles emerging from ASDs. They found that the increased permeation was independent of the ASD concentration. At higher ASD concentrations, where mixed micelles were observed, permeation enhancement was not measured. In addition, when micro-particles were removed, permeability was comparable to the crystalline drug. It can be hypothesized, that by removing the drug-rich particles, the molecularly dispersed concentration dropped to the level of the crystalline solubility, as the increased chemical potential of the amorphous form was missing to maintain the amorphous solubility. In addition to progesterone, a similar study was also carried out with rifaximin, a P-gp substrate, and different polymers (Beig et al., 2017). The authors found that with increasing apparent solubility, the absorption rate constant *in vivo* only increased only after passing a certain solubility threshold. They showed that this threshold is due to P-gp efflux saturation by inhibiting P-gp in the single-pass intestinal perfusion rat model.

A study by Ueda et al. compared the permeation of carbamazepine as ASDs, using HPMC-AS or Poloxamer 407 as the polymer component, based on dialysis membranes and Caco-2 monolayer experiments. Poloxamer 407 had a stronger solubilization effect on carbamazepine than HPMC AS. However, while HPMC-AS increased drug permeation, Poloxamer 407 decreased permeation. Authors postulated that HPMC-AS acts mainly as a crystallization inhibitor reducing the molecular mobility and that carbamazepine is self-associated in the HPMC-AS solution. In contrast to HPMC-AS, Poloxamer 407 formed micelles, encapsulating carbamazepine (Ueda et al., 2012). This underlines that these mechanisms of crystallization inhibition by polymers are advantageous compared to solubilization in micelles from the perspective of drug absorption. In addition, small drug-rich particles (<100 nm) could have an additional positive effect on bioavailability, mainly due to enhanced diffusion through unstirred layers (Kesisoglou et al., 2019).

In summary, drug absorption from dissolved ASD seems to be predominantly driven by a passive diffusion, which can be elevated by an increase in the concentration of molecularly dissolved API (e.g. by supersaturation), the limiting factor being the amorphous drug solubility. In contrast to other supersaturating (solubilizing) delivery systems, ASDs, with their ability to form drug-rich particles, show distinct advantages in mechanisms of increased bioavailability, mainly by elevating the mass transport through unstirred layers without negatively affecting the absorption.

### 7.2. Factors affecting drug uptake

Besides physicochemical considerations and the assumption of passive diffusion of API across the intestinal membrane, the physiology plays a significant role in drug uptake from ASDs. These factors add a greater level of complexity to the absorption mechanisms and are largely uninvestigated.

A study performed on curcumin as a model drug and rebaudioside A as carrier showed that ultra-small micelles (approximately 4 nm) are formed after dissolution of the solid system (Hou et al., 2019). Authors found indications for transcytosis of these particles in the everted intestinal ring model. Besides to energy-independent transport, authors also observed active transport. Furthermore, the particles have changed the intestinal areas of uptake to more proximal regions compared to the free drug. If latter was an effect of increased solubility and dissolution rate of the API or if it was related to their encapsulation into particles, remains an open question. A further increase in absorption can be triggered by the excipients themselves: a study analyzing ASDs with Soluplus as a polymer and lopinavir as a drug substance showed increased permeability *in vitro* and *in vivo* due to P-glycoprotein (P-gp) inhibition (Maibaum et al., 2004). Similarly, a study on ticagrelor ASD from TPGS and Neusilin^®^, a non-polymeric compound, reported an increased permeability through Caco-2 monolayers based on passive diffusion in addition to P-pg inhibition by TPGS. Also, increased bioavailability in rats was observed for this formulation (Kim et al., 2019). In a study by Huang et al. (2019), ASDs of curcumin prepared with chitosan oligosaccharides showed an increased permeability in a Caco-2 cell *in vitro* model and an increased bioavailability in rats. Authors attributed this effect mainly to an opening of the tight junctions in cell membranes by chitosan oligosaccharides. Furthermore, Girdhar et al. propose that for increased bioavailability, mucoadhesion is an essential factor to account for based on everted gut sac model experiments using dipyridamole. Mucoadhesive particles could locally increase the supersaturation close to the membrane and therefore increase the transmembrane flux (Girdhar et al., 2018).

Endogenous bile salts, as being surface-active substances, are expected to have an impact on ASD behavior. A study by Stewart et al. on itraconazole in HPMC-AS showed that with increasing bile-salt concentrations, the effect of drug-rich particles on improved permeation through the unstirred layer was diminished (Shi et al., 2015). At the same time, as discussed earlier (Section 6.2.3), bile salts can also have a stabilizing effect in supersaturated solutions.

Also, system approaches to elucidate mechanisms of increased bioavailability were published. Polster et al. observed a crystallization into another polymorph during the drug absorption process. Authors tested a formulation of LY2300599 in humans, showing an enhanced performance of the ASD formulation compared to a conventional formulation. In an artificial stomach duodenum model, authors characterized three steps leading to increased bioavailability: (1) rapid supersaturation in the stomach; (2) precipitation in the stomach into an amorphous solid; and (3) redissolution of the amorphous solid in the duodenum with supersaturated concentration levels. A special role was assigned to the excipient meglumine, as it was shown to facilitate drug dissolution by forming high pH regions which allow a poorly soluble weak acid to dissolve (Polster et al., 2015).

In conclusion, ASDs can also have physiological effects, such as inhibiting P-gp or affecting the tight junctions. Endogenous substances, such as bile salts, can have a direct impact on ASD performance. In addition, complex mechanisms from drug dissolution to drug uptake (e.g. temporary recrystallization) can occur, which makes rational development of ASDs a challenging task. Therefore, further research on these topics is necessary.

## 8. Equilibria and API distribution in dissolved ASDs

**Figure 5.**
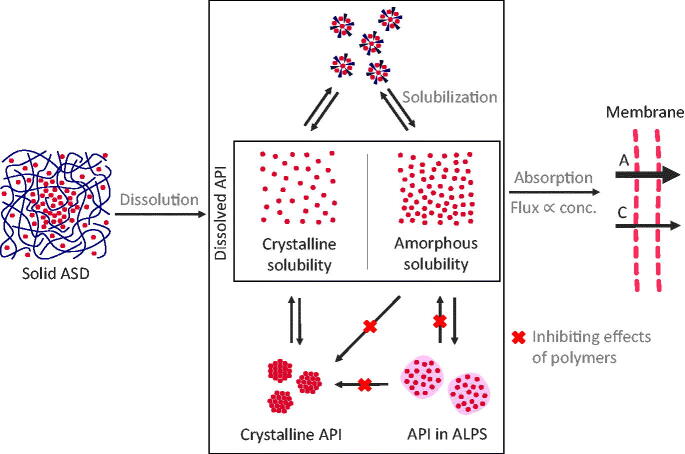
Scheme of the overall concept of increased bioavailability through ASDs. Upon dissolution of the solid ASD, a complex system of dissolved stated of ASDs emerges. In this system, solubilizing excipients can reduce the concentration of dissolved API. Supersaturation can be stabilized by polymers, however, polymers can also reduce the concentration of the molecularly dissolved API in favor of an increased fraction of ALPS. An increase in the molecularly dissolved drug concentration (conc.) from crystalline solubility (C) to amorphous solubility (A) leads to an increased transmembrane flux and therefore improved absorption.

For a complete mechanistic elucidation, it is important to understand also the equilibria of API distribution between the different states of dissolved ASDs, especially in the light of dynamic mechanisms of bioavailability, namely dissolution and absorption. As can be seen in Figure 5, we conceptualized three equilibria between (1) crystalline and molecularly dissolved API; (2) API in the ALPS phase and molecularly dissolved API (supersaturated); and (3) the solubilized API with the molecularly dissolved API. Drug absorption is proportional to the concentration of the drug in the molecularly dissolved state. The first equilibrium is well described and will therefore not be discussed here. The other equilibria, especially related to ASDs, will be discussed in this section.

Different research papers report the existence of equilibria between different dissolved states. Based on permeability results, Riana et al. showed a reduction of drug flux through membranes by surfactants, due to a dropped concentration of molecularly dissolved drug. They concluded that this effect can be compensated by increasing drug concentration in the system high enough that the molecularly dissolved API reaches the maximum ALPS concentration (saturation concentration of the solubilized state) (Stewart et al., 2017). A comparable idea of the equilibria between these states has been proposed by Fong et al. for solid phospholipid dispersions (Fong et al., 2016). This work supports the concept that there exist different states, i.e. the compartments solubilized API, molecularly dissolved API and API in ALPS, that have specific equilibrium coefficients and that the first two states can be saturated. From a drug delivery point of view, solubilization should not be a goal for formulation development, as solubilization could drain API from the molecularly dissolved state. This compartmental concept is also in line with the proposal that drug-rich particles from ASDs could represent a reservoir, from which API can be transferred rapidly into solution for subsequent absorption (Ilevbare & Taylor, 2013; Indulkar et al., 2017). Besides these three states, the polymers are assumed to not form an own state (except for micelle forming polymers). This is supported by a study from Deng et al. (2017) on dexamethasone crystallization inhibition by bile salts or lipid micelles solutions and its resulting effects on permeation. Authors revealed that the partitioning of the API into the micellar phase decreases permeation. This drug distribution was independent of the presence of the polymer (HPMC-AS). Authors concluded that drug solubilization in micelles and its crystallization inhibition by polymers can occur independently.

The equilibria are also dependent on other substances in the medium. A study by Ilevbare et al. (2013) investigated the impact of additives on the formation of drug-rich particles. Authors showed that additives (polymers and surfactants) influenced size, stability, and crystallization of the drug-rich particles. Charged additives inhibited droplet coalescence while their effect on crystallization was inconsistent, either showing promoting or inhibiting action. A study on paclitaxel in HPMC-AS showed that ALPS occurred at a lower concentration in the presence of polymer, indicating that the polymer was incorporated into the drug-rich particles. Authors assume that HPMC-AS is localized at the surface of formed drug-rich particles, as suggested by zeta-potential measurements (Miao et al., 2019). Furthermore, studies on multidrug ASDs (ritonavir, etravirine, and efavirenz in different cellulosic polymers) in solutions resulting from these ASDs showed that these substances influence each other in the measured aqueous concentration. Further mechanistic analysis lead to the conclusion that these interactions are not based on interactions within the molecularly dissolved state, but rather originate from interactions in the ALPS phase: the equilibrium between ALPS phase and the aqueous phase was shifted due to the intermixing of these drugs within the ALPS phase (Arca et al., 2017). In the case of polymers, the effect of their presence in drug-rich particles was investigated in more detail by the example of lopinavir: Li & Taylor (2018) conducted a study on the effect of polymers on supersaturation, differentiating by three levels of drug–polymer interaction. They pointed out that the amorphous solubility depends on the chemical potential within the amorphous phase. The admix of polymers alters the chemical potential of the amorphous phase. Based on theoretical considerations, authors propose three cases:

- Systems with stronger intermolecular interactions than within the pure components (negative Flory–Huggins interaction parameter). Here, an interaction of the API with the polymer is preferred in the amorphous phase, reducing the amorphous solubility.
- Systems with ideal intermolecular interactions (Flory–Huggins interaction parameters close to zero). Here, the change to the chemical potential is only related to the composition of the system (i.e. API concentrations in polymer and water).
- Systems with weaker intermolecular interactions (positive Flory–Huggins interaction parameter) than within pure components. Here, amorphous solubility is highest. However, these systems are prone to phase separation already in the solid ASD.

A negative effect on amorphous solubility is therefore expected for poorly soluble drugs, due to strong interactions with the poorly soluble API.

The proposed equilibria establish at a specific rate and are affected by dynamic factors. Consequently, sink conditions directly influence the *in vitro* and *in vivo* behavior of dissolved ASDs: A study (Bevernage et al., 2012) showed that the membrane transport of supersaturated loviride was reduced by crystallization and correlated with the degree of true supersaturation. On the other hand, crystallization was reduced by an absorptive environment. Therefore, the maximum absorption in an absorptive environment and in a non-absorptive environment did not correlate. The addition of crystallization inhibitors increased the membrane transport, however, significantly higher in a non-absorptive environment than in an absorptive environment.

In summary, the different states of dissolved ASDs can be seen as different compartments, namely the molecularly dissolved API, solubilized API, and the API in ALPS, which interact with each other and can reach saturation concentrations (except for API in ALPS). Polymers seem not to form their own state, but affect equilibria between the states. With respect to formulation development, the use of polymers should therefore be counterbalanced between strong interactions with the API to achieve efficient recrystallization inhibition (Section 6.2.2) and weak interactions to achieve maximal ALPS concentration for efficient trans-membrane flux. The effects of other substances (other drugs, food, or endogenous substances) on the equilibria underline the physicochemical sensitivity of the ALPS phase.

## 9. Conclusions and outlook

As outlined in this review article, recent literature contributes to a better understanding of mechanisms of ASD dissolution, drug distribution in an aqueous environment, and drug absorption. There has been compelling evidence collected that:

- ASD dissolution can be ascribed to three main mechanisms, namely carrier controlled, dissolution controlled, and drug controlled dissolution. Factors influencing drug dissolution are drug load, homogeneity of the solid ASD, drug–polymer interactions, and the presence of surfactants.
- The dissolved states of ASDs can be understood as states of molecularly dissolved drug (eventually supersaturated), solubilized drug, and drug in ALPS. These states are in a dynamic equilibrium.
- Increased drug absorption is mainly due to increased concentrations of molecularly dissolved API and facilitated diffusion through the unstirred layer by drug-rich particles. Effective release is from the drug-rich particles is a distinct advantage over other enabling formulation for poorly soluble APIs.
- Polymers can stabilize supersaturated solutions in the aqueous and the amorphous phase. At the same time, however, they can lower the amorphous solubility.
- Surfactants can enhance dissolution properties, stabilize but also destabilize supersaturated solutions and decrease the dissolved fraction of API when forming a micellar state.

Translation from *in vitro* to *in vivo* remains a challenge. There are only a few studies, which address this problem. For example, physiologically based pharmacokinetic (PBPK) modeling or IVIVC (*in vitro*–*in vivo* correlation) strategies were used to predict *in vivo* performance of ASDs (Sethia & Squillante, 2004; Indulkar et al., 2016). We believe that translational approaches could greatly benefit from a better mechanistic understanding of underlying processes of API delivery, i.e. liberation, and absorption in biological systems.
